# Phospholipase A_2_ Isolated from the Venom of *Crotalus durissus terrificus* Inactivates *Dengue virus* and Other Enveloped Viruses by Disrupting the Viral Envelope

**DOI:** 10.1371/journal.pone.0112351

**Published:** 2014-11-10

**Authors:** Vanessa Danielle Muller, Ricardo Oliveira Soares, Nilton Nascimento dos Santos-Junior, Amanda Cristina Trabuco, Adelia Cristina Cintra, Luiz Tadeu Figueiredo, Antonio Caliri, Suely Vilela Sampaio, Victor Hugo Aquino

**Affiliations:** 1 Laboratório de Virologia, Departamento de Análises Clínicas, Toxicológicas e Bromatológicas, Faculdade de Ciências Farmacêuticas de Ribeirão Preto, Universidade de São Paulo, Ribeirão Preto, São Paulo, Brasil; 2 Laboratório de Toxinologia, Departamento de Análises Clínicas, Toxicológicas e Bromatológicas, Faculdade de Ciências Farmacêuticas de Ribeirão Preto, Universidade de São Paulo, Ribeirão Preto, São Paulo, Brasil; 3 Departamento de Física e Química, Faculdade de Ciências Farmacêuticas de Ribeirão Preto, Universidade de São Paulo, Ribeirão Preto, São Paulo, Brasil; 4 Centro de Pesquisa em Virologia, Departamento de Clínica Médica, Faculdade de Medicina de Ribeirão Preto, Universidade de São Paulo, Ribeirão Preto, São Paulo, Brasil; Universidad de Costa Rica, Costa Rica

## Abstract

The *Flaviviridae* family includes several virus pathogens associated with human diseases worldwide. Within this family, *Dengue virus* is the most serious threat to public health, especially in tropical and sub-tropical regions of the world. Currently, there are no vaccines or specific antiviral drugs against *Dengue virus* or against most of the viruses of this family. Therefore, the development of vaccines and the discovery of therapeutic compounds against the medically most important flaviviruses remain a global public health priority. We previously showed that phospholipase A_2_ isolated from the venom of *Crotalus durissus terrificus* was able to inhibit *Dengue virus* and *Yellow fever virus* infection in Vero cells. Here, we present evidence that phospholipase A_2_ has a direct effect on *Dengue virus* particles, inducing a partial exposure of genomic RNA, which strongly suggests inhibition via the cleavage of glycerophospholipids at the virus lipid bilayer envelope. This cleavage might induce a disruption of the lipid bilayer that causes a destabilization of the E proteins on the virus surface, resulting in inactivation. We show by computational analysis that phospholipase A_2_ might gain access to the *Dengue virus* lipid bilayer through the pores found on each of the twenty 3-fold vertices of the E protein shell on the virus surface. In addition, phospholipase A_2_ is able to inactivate other enveloped viruses, highlighting its potential as a natural product lead for developing broad-spectrum antiviral drugs.

## Introduction

The *Flaviviridae* family includes several virus pathogens associated with human diseases worldwide. Clinical conditions can vary from febrile or hemorrhagic diseases for *Dengue virus* (DENV) and *Yellow fever virus* (YFV), encephalitis for *Saint Louis encephalitis virus* (SLEV), *Japanese encephalitis virus* (JEV), *Tick-borne encephalitis virus* (TBEV), *West Nile virus* (WNV), and *Rocio virus* (ROCV) to hepatitis for *Human hepatitis C virus* (HCV*)* and *Human Pegivirus* (HPgV). Currently, preventative vaccines for humans are available only for YFV, TBEV, and JEV and specific antiviral treatment only for HCV [1]. Therefore, the development of vaccines and the discovery of therapeutic compounds against the medically most important flaviviruses remain a global public health priority [2].

Of the diseases caused by viruses of the *Flaviviridae* family, dengue is a major threat to public health. It is estimated that 390 million dengue infections occur per year, with 100 million manifesting some type of symptoms [3] and approximately two million requiring hospitalization [4]–[6]. The major goal of anti-DENV therapy is to prevent patients from developing the severe forms of the disease [7].

Members of the *Flaviviridae* family include viruses with a positive-sense, single-stranded RNA genome of approximately 11,000 nucleotides, surrounded by a nucleocapsid and covered by a lipid envelope in which viral glycoproteins are anchored. The RNA genome encodes a single polyprotein that is proteolytically cleaved into three structural proteins (C-prM/M-E) and seven non-structural proteins (NS1-NS2A-NS2B-NS3-NS4A-NS4B-NS5) [8].

Natural products offer a huge amount of compounds with a great diversity of chemical structures, the result of biosynthetic processes that have been modulated over millennia through evolution. Natural products have served as important sources of drugs for medical purposes. Tubocurarine, a toxic alkaloid with skeletal muscle relaxant properties and obtained from the bark of the South American plant *Chondrodendron tomentosum*, was the first naturally occurring toxin used in medicine [9]. Since 1970, more than 40 drugs derived from natural products have been approved for use in humans [10]–[12]. Among natural products, venoms are complex mixtures of many different components, such as metalloproteinases, serine proteinases, potassium channel-binding neurotoxins, proteolytic enzymes, cytotoxins, pre- and post-synaptic neurotoxins, cardiotoxins, and phospholipase A_2_s, which can provide clues for designing therapeutically useful molecules [13]. Indeed, Ferreira *et al.* [14] provided a good example of the potential of snake venom components for successful drug development. These authors identified bradykinin-potentiating peptides from the Brazilian arrowhead viper (*Bothrops jararaca*) venom that were used to develop an inhibitor (captopril) of the angiotensin-converting enzyme that is widely used as an anti-hypertensive agent [15]. Two other drugs, Tirofiban and Eptifibatide, were designed based on snake venom components and are available in the market as antiplatelet agents [16], [17]. In addition, snake venoms and their components have shown antiviral activity against *Measles virus* [18], *Sendai virus* [19], *Dengue virus* [20], [21], and *Human immunodeficiency virus* (HIV) [22]. One of the main components of snake venom is secreted phospholipase A_2_ (sPLA_2_), which has shown systemic toxicities that include myotoxicity, cardiotoxicity, neurotoxicity, nephrotoxicity, hepatotoxicity, reprotoxicity, and systemic hemorrhage [23]–[28]. The sPLA_2_ isolated from snake venoms and other sources has also shown antiviral activity against HIV [22], [29], [30], adenovirus [31], *Newcastle virus* [32], and *Rous virus* [33]. In addition, we have recently found a high antiviral effect of sPLA_2_ (crotoxin, a dimeric compound composed of PLA_2_-CB and crotapotin, isolated PLA_2_-CB and PLA_2_-inter-cro) from *Crotalus durissus terrificus* venom against DENV and YFV [21]. In this study, we further analyzed the antiviral effect of PLA_2_-CB against dengue virus and three other enveloped viruses.

## Materials and Methods

### Cells and viruses

VERO E6 (African green monkeys kidney epithelium cell line) [34] and C6/36 (*Aedes albopictus* mosquito cell line) [35] cells were maintained in Leibovitz's medium (L-15) with 10% fetal bovine serum (FBS) at 37°C and 28°C, respectively. DENV-2 (NGC strain), *Rocio virus* (SPH 34675 strain), *Mayaro virus* (BeAn 243 strain), and *Oropouche virus* (BeAn 19991 strain) from the virus collection of the Virology Research Center of the Medical School of Ribeirao Preto, University of Sao Paulo, were used in this study. Enterovirus *Coxsackie-B5 virus* was kindly provided by Prof. Dr. Eurico Arruda Neto from the Viral Pathogenesis laboratory at the Virology Research Center, Medical School of Ribeirao Preto, University of Sao Paulo. Virus titration was performed in the Vero E6 cell line using the plaque assay, and the titer was expressed in plaque-forming units per milliliters (PFU/mL), as previously described [21].

### *Dengue virus* RNA detection and quantification: Quantitative real-time RT-PCR (qRT-PCR)

Viral RNA detection was carried out by real-time RT-PCR using the SuperScript III Platinum SYBR Green One-Step qRT-PCR kit (Invitrogen, USA), as previously described [36]. Briefly, the 25-µL reaction mixture contained 0.5 µL of SuperScript III RT Platinum Taq Mix, 0.2 mM of each primer, 12.5 µL of 2× SYBR Green, and 5 µL of purified RNA. The amplification program was as follows: 50°C for 20 min for reverse transcription, 95°C for 5 min for reverse transcriptase inhibition and *Taq* DNA polymerase activation, followed by 45 cycles of PCR amplification with denaturation at 95°C for 15 sec, annealing at 60°C for 40 sec, and extension at 72°C for 30 sec. Finally, to verify the specificity of the PCR products, a melting curve was constructed by incubating the amplification products from 60°C to 90°C with an increase of 0.2°C/sec. The melting temperature (Tm) values of the specific amplicons were in the range of 80.57°C-81.73°C. For viral load determination, a standard curve was constructed using RNA transcribed *in vitro* from a plasmidial clone containing a fragment of 2500 base pairs (bp) corresponding to the 5′ end of DENV-3 strain D3BR/RP1/2003 [37]. This plasmid was prepared as follows: the 2500-bp fragment was amplified by RT-PCR and inserted into the plasmid pXL (Invitrogen, USA), which was used to transform *Escherichia coli*. An aliquot of 250 µL of the bacteria was inoculated into 10 mL of LB+ampicillin medium, followed by incubation at 37°C for 14 h with shaking. The plasmid was purified using the QIAGEN Plasmid Mini Kit (Qiagen, Germany) following the manufacturer's recommendations. The plasmid was linearized by digestion with Bam HI, subjected to electrophoresis through a 1% agarose gel, and purified from the gel using the QIAquick Gel Extraction Kit (Qiagen, Germany) following the manufacturer's specifications. For RNA preparation, 1 µg of the linearized plasmid was transcribed using RNAMaxx High Yield Transcription Kit (Stratagene, USA) following the manufacturer's specifications. The cDNA was digested using RQ DNase (Promega, USA) at 37°C for 20 min. After this, the RNA was purified using the QIAamp Viral RNA Kit (QIAGEN, Germany), and the concentration was determined by spectrophotometry at 260 nm. Based on the concentration and size of the RNA product (2500 bp), the copy number equivalent to the genomic RNA was calculated. Serial decimal dilutions of this RNA were prepared to construct the standard curve by real-time RT-PCR for determination of the virus titer, which was expressed in copy numbers per milliliters.

### Venom and toxin purification from *Crotalus durissus terrificus*

Snake venom was obtained from the serpentarium of the Medical School of Ribeirao Preto, University of Sao Paulo (authorization of the *Instituto Brasileiro do Meio Ambiente e dos Recursos Naturais Renováveis*, IBAMA: 1/35/1998/000846-1). Purification and evaluation of the enzymatic activity of PLA_2_-CB and crotoxin were carried out as previously described [21].

### Antiviral assays of PLA_2_-CB and crotoxin against DENV-2

#### Virucidal assay: virus treatment before infection

To evaluate the direct effect of PLA_2_-CB and crotoxin on viral particles, DENV-2 (2×10^3^ PFU) was incubated with non-cytotoxic concentrations (8, 4, and 0.004 ng/µL) of PLA_2_-CB and crotoxin in a final volume of 100 µL for 1 h at 37°C [21]. DENV-2 incubated with PBS was used as a control. The mixtures were diluted 100 times with L-15 serum-free medium and added to VERO E6 cells seeded in 24-well plates (2×10^5^ cells/well). After dilution, two concentrations (0.008 and 0.004 ng/µL) were above and one bellow (0.000004 ng/µL) the effective concentration that inhibits 50% (EC_50_) of cells infection found in our previous study (EC_50_ of 0.00003 for PLA_2_-CB and 0.001 for crotoxin) [21]. After 1 h of incubation at 37°C, the supernatant was removed, and 1 mL of L-15 medium supplemented with 2% fetal bovine serum (FBS) was added to the cells, followed by a further incubation at 37°C for 72 h. The cell culture supernatants were collected for viral inhibition evaluation by qRT-PCR, as mentioned above.

#### Pre-treatment assay: Treatment of cells with toxins before virus infection

This assay was carried out to evaluate whether the toxins could protect VERO E6 cells against viral infection. VERO E6 cells were seeded (2×10^5^ cells/well) in a 24-wellsplate and incubated at 37°C for 24 h. The medium was then removed, and the cells were incubated with several concentrations of PLA_2_-CB or crotoxin (0.000004, 0.04, 0.08, 0.5, 1, and 2 ng/µL). After 1 h of incubation, the supernatant was removed, and cells were washed five times with PBS at room temperature. The cells were infected with 25–100 PFU of DENV-2 in 400 µL of L-15 for 1 h. The supernatant was removed, and 1 mL of L-15m supplemented with 2% FBS was added to the cells. After 72 h of incubation at 37°C, the cell supernatants were collected for viral RNA extraction using AxyPrep Body Fluid Viral DNA/RNA Miniprep Kit (Axygen, USA) following the manufacturer's recommendations. Virus inhibition was evaluated by qRT-PCR, as mentioned above.

### Evaluation of DENV-2 RNA exposure induced by PLA2-CB and crotoxin

This assay was carried out as previously proposed [38]. DENV-2 (2.0×10^3^ PFU) was incubated with 8 ng/µL of PLA_2_-CB, 8 ng/µL of crotoxin, 1 µg/µL of proteinase K (Life Technologies, USA), 1% Triton X-100 (Life Technologies, USA), or PBS in a total volume of 100 µL for 1 h and 2 h at 37°C. Then, the virus was treated with PBS or RNase A (Promega, USA) according to the manufacturer's recommendation. RNA purified from DENV-2 (2.0×10^3^ PFU) was used as a control for RNA degradation induced by RNase A. After 1 h at 37°C, the viral RNA was extracted using AxyPrep Body Fluid Viral DNA/RNA Miniprep Kit (Axygen, USA), and RNA degradation was evaluated by qRT-PCR as mentioned above.

### Virucidal assay of PLA2-CB and crotoxin against other enveloped and non-enveloped viruses

The enveloped viruses *Dengue virus* type 2, *Rocio virus*, *Mayaro virus*, and *Oropouche virus* and the non-enveloped *Coxsackie B5 virus* were used in this experiment. The viruses (25–100 PFU) were incubated with serial decimal dilutions of PLA_2_-CB and crotoxin (50, 5, 0.5, 0.005, 0.0005, and 0.00005 ng/µL) for 1 h at 37°C. The treated viruses were used to infect VERO E6 cells contained in a 24-well plate for 1 h. The supernatant was removed, and the cells were overlaid with 1 mL of L-15 supplemented with 2% FBS and 1.8% carboxymethylcellulose. After 7 days at 37°C, the semisolid overlay medium was removed, and the cells were fixed and stained with naphthol blue-black in 5% acetic acid for plaque counting. The treatment of each virus was repeated three times, and the concentration that inhibited 50% of plaque formation (Effective Concentration 50%, EC_50_) was calculated by comparison with the number of plaques observed in the PBS-treated cells.

### Steric and electrostatic analysis of the interaction of PLA2-CB with the lipid bilayer of the DENV envelope

The DENV external structural model was obtained from RCSB Protein Data Bank under the PDB ID: 3J35 [39]. This model was developed by Zhang *et al.* [40] by electron microscopy analysis and reflects the structure of DENV under physiological temperature (37°C), in accordance with the experiments conducted in this work. We used this electron microscopy structure as a model to reconstruct a more refined version of the virus structure, fitting 180 high-resolution copies of DENV-2 E protein (PDB ID: 1OAN) [41]. The DENV membrane patch used to reproduce the 3-fold pore environment in the electrostatic analyses was assembled with the CHARMM-GUI program [42] and further thermalized with the GROMACS Molecular Dynamics package [43]; the lipid composition follows that described by Zhang *et al.* [44]. The resolved PLA_2_-CB crystal structure was obtained under PDB ID: 2QOG [45]. Because this model is built as a tetramer, we extracted one monomer (including the Ca^2+^ ion) with software Visual Molecular Dynamics – VMD [46], completing the few missing atoms from residues Trp31 and Pro74 with the aid of the PyMol program [47], and finally performed an adjustment with a potential energy minimization of the side chains with the GROMACS Molecular Dynamics package [43] using the IBM BlueGene/P supercomputer hosted at Rice University. For the all-atom MD with the explicit solvent [TIP3P water model [48] constrained by the SETTLE algorithm [49]], we used the CHARMM36 force-field [50] adapted to GROMACS by Piggot and colleagues, 2012 [51], with the Particle Mesh Ewald (PME) treatment for the electrostatic interactions among the atoms, with a cut-off of non-bonded interactions of 1.0 nm. The neutral pH of the system was emulated indirectly by fixing the protonation states of ionizable side chains at their correspondent pKa values, according to Nozaki and Tanford [52]. The temperature of the system was set to 310 K, and the pressure was maintained at 1 ATM using the Nose-Hoover and Parrinello-Rahman algorithms, respectively [53]–[55]. The simulation protocol starts with potential energy minimizations with the steep descent method until the convergence of an energy threshold smaller than 2×10^3^kJ mol^−1^ nm^−1^. Maxwell-Boltzmann distribution assigns velocities to all atoms in the system according to the chosen initial temperature, and a position restriction was applied to the protein portion, allowing full solvation with the newly introduced water. After this step, we removed all restrains from the heavy atoms of the protein and used the P-LINCS algorithm [56] to constrain all bonded interactions, allowing us to set a time step of 2 femtoseconds. The electrostatic analyses and electrical field of the system consisting of one monomer of PLA_2_-CB plus the extracted 3-fold pore from DENV were generated with the APBS (Adaptive Poisson-Boltzmann Solver) plugin [57] in the PyMol software [47]. The atomic individual charges of PLA_2_-CB and the DENV 3-fold pore were attributed with aid of the pdb2pqr online server [58], and their protonation states were determined at pH 7 by PROPKA 3.1 [59]. The charges from the membrane atoms were calculated and assigned with the program *editconf* (-mead option) in the package GROMACS 4.5.5. In all cases, we set as default the solvent and protein/lipid dielectric constants to 78.0 and 2.0, respectively.

### Statistics

A Mann–Whitney U test was used to compare the differences between the treated and untreated control groups. *P* values of <0.05 were considered to indicate statistically significant differences. The statistical analyses were carried out using GraphPad Prism version 5.03 (GraphPad Software, La Jolla, CA).

## Results

### Evaluation of the direct action of PLA2-CB and crotoxin on the DENV-2 virion: Virucidal assay

**Figure 1:**
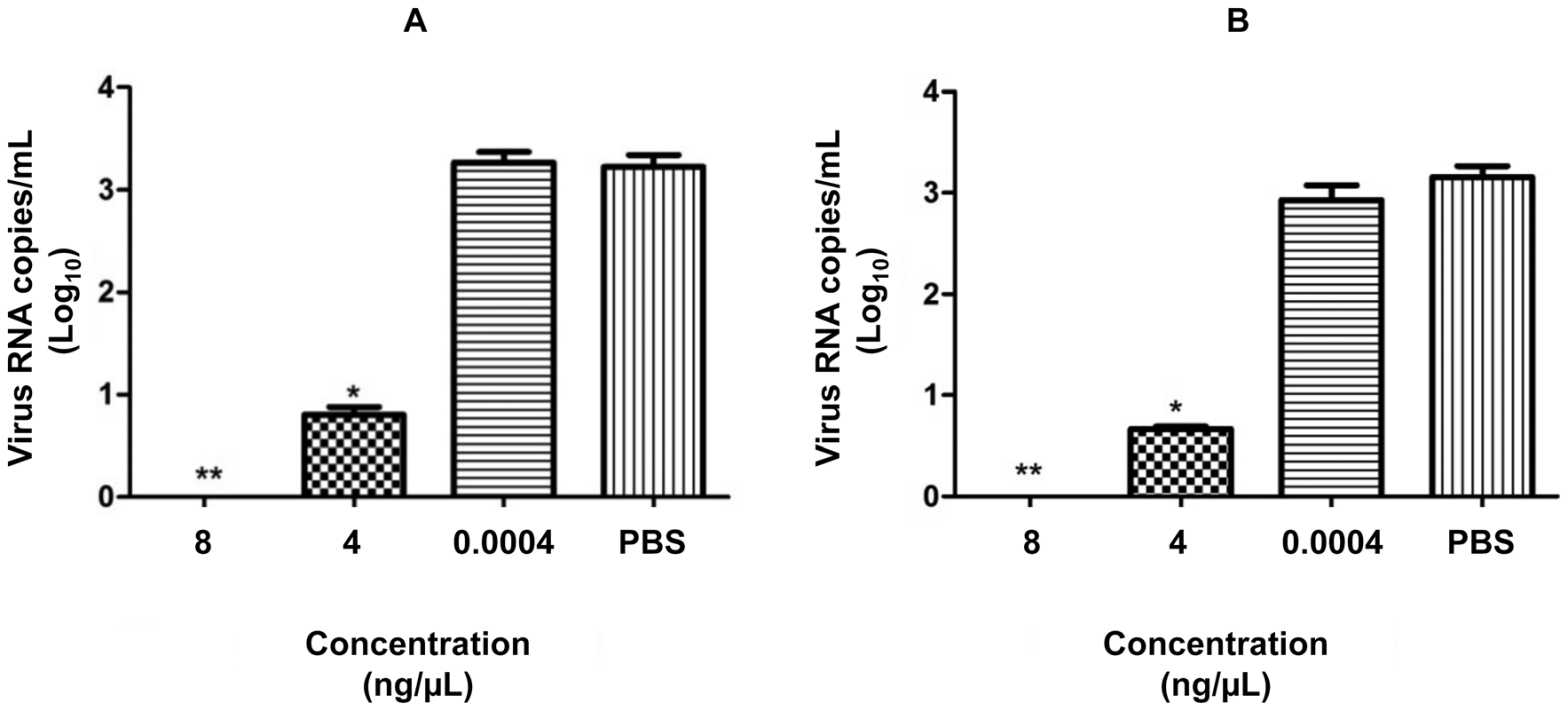
Virucidal assay. DENV-2 was treated with different concentrations of PLA_2_-CB (a) and crotoxin (b) and then used to infect Vero cells for 72 h. The antiviral effect of the toxins was evaluated by determining the virus titer in the cell culture supernatant by qRT-PCR. The data represent mean values ± standard deviations (SD) for three independent experiments. The asterisks indicate statistically significant differences from PBS-treated viruses (*p<0.05, **p<0.01).

To analyze whether PLA_2_-CB and crotoxin have a direct effect on the virus particle, DENV-2 (2.0×10^3^ PFU) was treated with 8, 4, and 0.0004 ng/µL of each toxin or PBS. One hour after treatment, the mixture was diluted 100 times (final concentration 0.08, 0.04, and 0.000004 ng/µL of toxins and 20 PFU of DENV-2) and then used to infect Vero cells. The purpose of the dilution was to reduce the concentration of toxins in contact with the cells and to use concentrations above (0.08 and 0.04 ng/µL) and below (0.000004 ng/µL) the EC_50_ found in our previous study [21]. Viral replication was evaluated in the cell culture supernatant after 72 h of infection by qRT-PCR. A significant inhibition of virus replication was observed when the virus was treated with 8 and 4 ng/µL of PLA_2_-CB and crotoxin in a dose-dependent manner, suggesting a direct action of the toxins on the virus particle (Figure 1). However, despite the low concentration of toxins, some effect on Vero cells that trigger an antiviral mechanism cannot be discarded.

### Pre-treatment assay: Treatment of cells with toxins before virus infection

**Figure 2:**
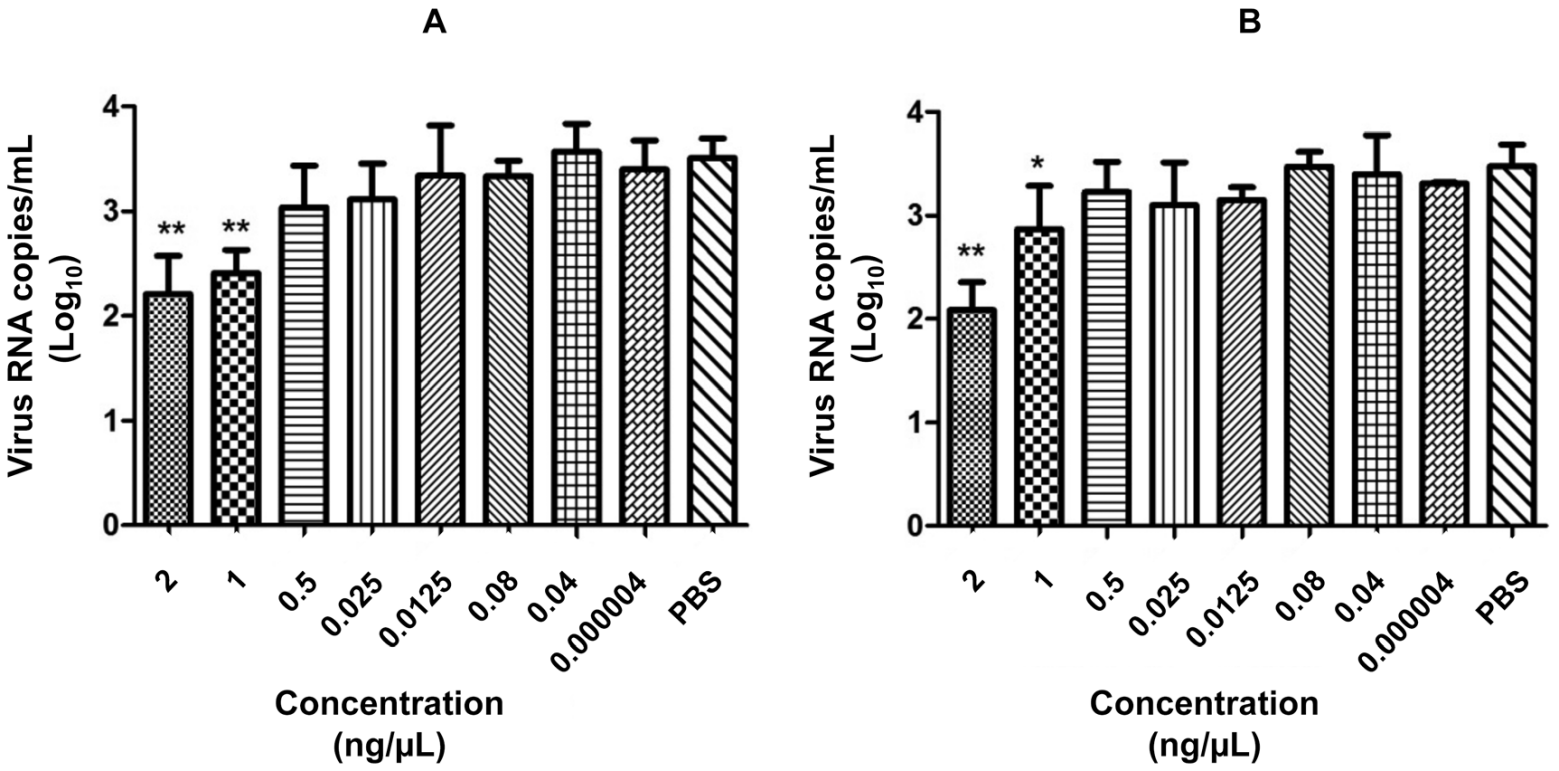
Pre-treatment assay. Vero cells were treated with different concentrations of PLA_2_-CB (a) and crotoxin (b) and then infected with DENV-2 for 72 h. The antiviral effect of the toxins was evaluated by determining the virus titer in the cell culture supernatant by qRT-PCR. The data represent mean values ± standard deviations (SD) for three independent experiments. The asterisks indicate statistically significant differences from PBS-treated cells (*p<0.05, **p<0.01).

To better analyze whether PLA_2_-CB and crotoxin have some effect on Vero cells that influences DENV-2 inhibition, the cells were treated with several concentrations of PLA_2_-CB and crotoxin, including those used in the virucidal assay after dilution (0.000004, 0.04, 0.08, 0.5, 1, and 2 ng/µL), before infection. One hour after treatment, the cells were washed and infected with the same amount of DENV-2 used in the virucidal assay (20 PFU). The qRT-PCR analysis of the cell culture supernatant after 72 h of infection showed that virus replication was significantly inhibited only at the higher toxin concentrations, 1 and 2 ng/µL (Figure 2). This result strongly suggests that the antiviral effect of PLA_2_-CB and crotoxin observed in the virucidal assay was due to a direct action on the virus particle.

### *In vitro* DENV-2 RNA exposure induced by PLA2-CB and crotoxin

**Figure 3:**
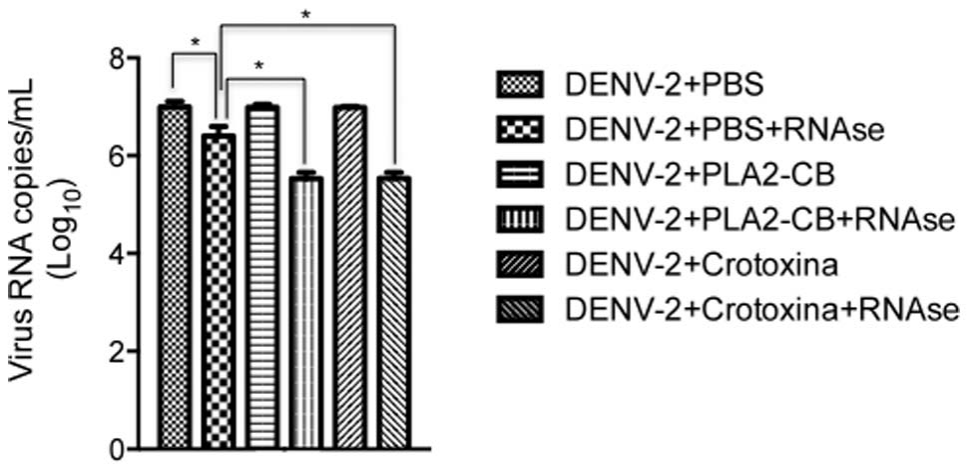
Analysis of the exposure of DENV-2 genomic RNA. DENV-2 was first treated with PLA_2_-CB, crotoxin (8 ng/µL each) or PBS at 37°C and then with RNase-A. Virus RNA degradation was evaluated by qRT-PCR. The data represent mean values ± standard deviations (SD) for three independent experiments. The asterisks indicate statistically significant differences among groups (*p<0.05).

**Figure 4:**
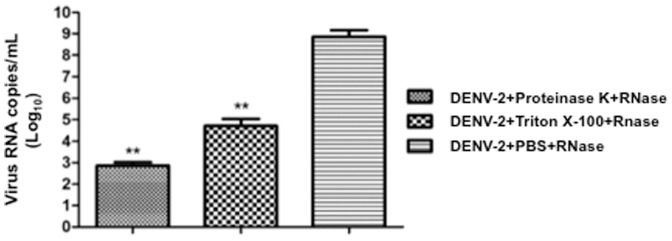
Analysis of the exposure of DENV-2 RNA. DENV-2 was first treated with proteinase K, Triton X-100 and PBS and then with RNase-A. Virus RNA degradation was evaluated by qRT-PCR. The data represent mean values ± standard deviations (SD) for three independent experiments. The asterisks indicate statistically significant differences from PBS-treated viruses (**p<0.01).

The antiviral effect of toxins might be related to enzymatic activity by means of glycerophospholipid cleavage at the virus lipidic envelope, which could induce an exposure of the virus RNA genome. To investigate this hypothesis, DENV-2 was treated with 8 ng/µL of PLA_2_-CB and crotoxin for 1 and 2 hours at 37°C and then with RNAse-A to evaluate RNA exposure. A significant viral RNA degradation was observed when DENV-2 was treated with PLA_2_-CB and crotoxin, suggesting virus RNA exposure (Figure 3). However, increased RNA degradation was observed when DENV-2 was previously treated with protease (proteinase K) or detergent (Triton X-100), both of which destroy the virus envelope and induce the complete exposure of genomic RNA (Figure 4). Therefore, the lower level of genomic RNA degradation observed after PLA2-CB or crotoxin treatment suggests a partial exposure of the virus RNA by the partial disruption of the virus envelope. Incubation of DENV-2 with toxins for more than 1 hour did not increase the viral RNA degradation, suggesting that all the available glycerophospholipid on the virus envelope were already cleaved in 1 hour.

### Evaluation of PLA2-CB and crotoxin antiviral activity against other enveloped viruses

The above experiments suggested that PLA_2_-CB and crotoxin exert their antiviral functions via partial cleavage of the virus envelope, which has a cellular origin. Therefore, these toxins could also have an antiviral effect against other enveloped viruses. To test this hypothesis, we evaluated the antiviral activity of PLA_2_-CB and crotoxin, in a virucidal assay, against other enveloped viruses, *Rocio virus* (*Flaviviridae* family), *Oropouche virus* (*Bunyaviridae* family), and *Mayaro virus* (*Togaviridae* family) and the non-enveloped virus *Coxsackie B5 virus* (*Picornaviridae* family). As expected, the toxins showed a high antiviral effect (very low EC_50_) only against the enveloped viruses (Table 1, Figure S1–S8).

**Table 1:** Virucidal assay.

Virus	Toxin	EC_50_ (ng/µL)	95% confident limit (ng/µL)
***Dengue virus*** ** type 2**	PLA_2_-CB	6.6E-5	2.56E-5-1.78E-4
	Crotoxin	0.0078	0.0042–0.015
***Rocio virus***	PLA_2_-CB	0.021	0.0095–0.048
	Crotoxin	0.0046	0.0029–0.0074
***Mayaro virus***	PLA_2_-CB	0.066	0.025–0.18
	Crotoxin	0.0036	0.0012–0.011
***Oropouche virus***	PLA_2_-CB	0.0067	0.0023–0/019
	Crotoxin	0.0054	0.0023–0.013
***Coxsackie B5 virus***	PLA_2_-CB	-	
	Crotoxin	-	

Antiviral activity of PLA_2_-CB and crotoxin against Dengue virus type 2, Rocio virus, Mayaro virus, Oropouche virus, and Coxsackie B5 virus.

### Steric and electrostatic analysis of the interaction of PLA_2_-CB with the DENV envelope lipid bilayer

**Figure 5:**
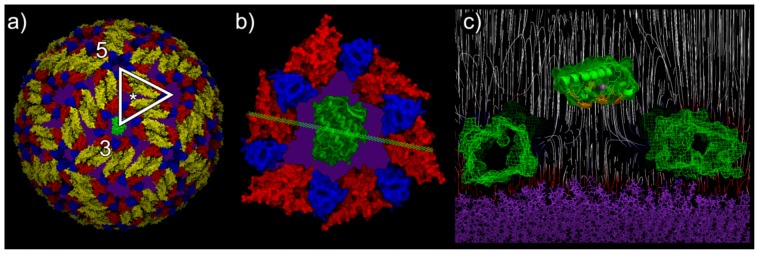
Structural analyses of DENV particle and PLA_2_-CB. **a**) View of DENV at physiological temperature with one PLA_2_-CB molecule shown at the putative site of interaction. The triangle shows one unit of the icosahedral symmetry, with the 5-fold and 3-fold vertices and intermediary areas indicated with an asterisk. The E protein domains I, II, and III are colored red, yellow, and blue, respectively; exposed membrane patches are colored in purple. **b**) Detailed view of the PLA_2_-CB/DENV 3-fold pore complex. The green line marks the slab view on **c**. **c**) Electric field lines of the PLA_2_-CB/DENV 3-fold pore complex; the pore domains are represented on both sides in green mesh surface, whereas PLA_2_-CB is located in the middle. Hydrophobic aromatic residues in the i-face are colored in orange; the Ca^2+^ ion is colored in light purple. The membrane is colored in purple; blue and red lines indicate the proximity of positive and negative charges, respectively.

The results presented thus far consistently indicate an effective selectivity of PLA2-CB and crotoxin action in inhibiting enveloped viruses. By including structural analyses, we gained a more comprehensive knowledge of such selectivity and its process by outlining the implicated molecular mechanism of the PLA_2_-CB/crotoxin–DENV interaction. The results concerning the non-enveloped *Coxsackie virus*, in addition to the similar success rates shared between DENV and other enveloped viruses in virucidal assays, suggest a direct catalytic action on the phospholipid bilayer. In DENV, however, this structure is not fully exposed: it lies below the protein E shell, which must be surmounted by PLA2-CB to gain access to its substrate. At physiological temperature (37°C), *Dengue virus* assumes an expanded and mostly spherical configuration, whereby E protein sub-units are arranged in such a conformation that leads to an enhanced area of lipid envelope exposure [40]. Although essentially spherical, this configuration inherits the characteristic icosahedral symmetry from the more compact configuration at room temperature and can be described as a function of it. Three repeating sets of major gaps among the E proteins were identified in the 3-fold axis pores [60], the 5-fold axis pores, and the middle points among them (Fig. 5, a). However, cryo-electron microscopy analyses showed that the widest areas of exposure are those located on each of the twenty 3-fold axes [44]. These areas comprise the smaller radial distances from the center of the virus (∼232 Å^2^, as opposed to the highest value of ∼264 Å^2^), indicating that these regions concentrate the deepest cavities on the virus surface. Combining the properties of this pronounced cavity with its aforementioned large diameter, it becomes clear that the 3-fold pore is indeed a remarkable site of exposure of the buried DENV lipid envelope. The planar surface that surrounds the active site (i-face) from the bovine group-IB (bGIB) PLA_2_ has an area of approximately 1500 Å^2^ [61]; because snake PLA_2_-CB shares a very similar structure with (bGIB) PLA_2_, we assume the same i-face area for both. By structural analysis, we estimated that the 3-fold pore has an area of at least approximately 2500 Å^2^, being therefore at least 60% wider than a PLA_2_-CB monomer by superimposition at the planar orientation (Fig. 5, b). Such orientation is primarily based on taking full advantage of the interaction of the hydrophobic amino acids of PLA_2_-CB (highlighting Trp31, Trp70, Tyr73, and Phe119) with the hydrophobic matrix just below the level of the polar phospholipid heads in the zwitterionic DENV bilayer. Electrostatic analyses of the complex PLA_2_-CB/DENV 3-fold pore showed that the prominently positive inner surface of the 3-fold pore facing the negative charges of the membrane tends to establish a perpendicular electrical field to the plane of membrane around its periphery, whereas the centermost region displays a weaker electrical field (Fig. 5, c). We believe that because PLA_2_-CB displays a net positive surface charge, the particular setting of these fields could slightly repel it from the pore edges. The combined repulsion of the pore edges maintains PLA_2_-CB in the central more neutral field; at the same time, the membrane polarity could play an increasing attracting role as their distance reduces. Once PLA_2_-CB reaches the membrane, it establishes contact with the phospholipids, which are hydrolyzed, destabilizing the membrane and therefore the general integrity of the virus.

**Figure 6:**
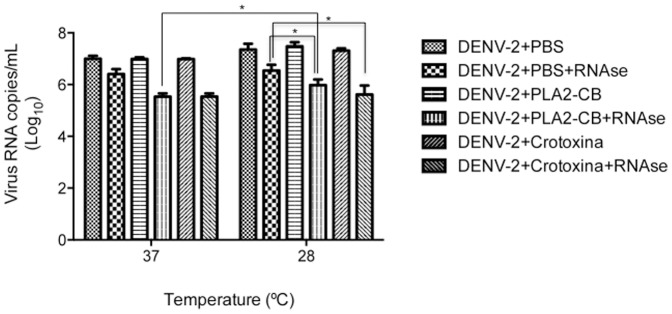
Analysis of the exposure of DENV-2 RNA. DENV-2 was first treated with PLA_2_-CB, crotoxin (8 ng/µL each) and PBS at 37°C and 28°C and then with RNase-A. Virus RNA degradation was evaluated by qRT-PCR. The data represent mean values ± standard deviations (SD) for three independent experiments. The asterisks indicate statistically significant differences among groups (*p<0.05).

To further analyze if the virus external structure influence the antiviral activity of toxins, and considering that DENV acquire a “bumpy,” expanded conformation when incubated at 37°C as opposed to its smooth structure at room temperature [40], DENV-2 was incubated with toxins at 28°C (temperature used to infect the mosquito C6/36 cells) and 37°C and then treated with RNase (Figure 6). A significant higher RNA degradation was observed when DENV-2 was treated with PLA_2_-CB at 37°C, supporting the hypothesis that at this temperature the relaxed viral external structure allows an enhanced contact of PLA_2_-CB with the glycerophospholipid on the virus envelope. However, the level of RNA degradation was the same at both temperatures for crotoxin. The last result might be related to the heterodimeric nature of this toxin (PLA_2_-CB+crotapotin); crotapotin act as a chaperon that enhances PLA_2_-CB activity [62].

## Discussion

In a previous study, we showed that PLA_2_s isolated from the venom of *Crotalus durissus terrificus* have a potent antiviral action against DENV-2 and YFV, especially at the early stage of the multiplication cycle [21]. Inhibition of the pathogen infectivity induced by PLA_2_ can occur by direct action on the pathogen or as alternative processes on the host or surrounding medium [63]–[65]. Here, we demonstrate a dose-dependent inhibition of DENV-2 infection in Vero cells induced by crotoxin and PLA_2_-CB in a virucidal assay, providing strong evidence of the direct action of these toxins on the virus particle. Crotoxin is a heterodimeric protein composed of the basic PLA_2_-CB and the acidic nontoxic catalytically inactive protein crotapotin, which act as a chaperon enhancing PLA_2_-CB activity [62]. Therefore, virus inhibition is most likely induced by the PLA_2_-CB enzymatic activity, which relies on the hydrolysis of the sn-2 acyl ester linkage of sn-3-glycero-phospholipids, producing fatty acids and lysophospholipids as reaction products [66]. In accordance with this hypothesis, we have found previously, performing an virucidal assay, that PLA_2_-CB lost its antiviral activity in the absence of Ca+2, which is an essential cofactor for its enzymatic activity, or by the inhibition of the enzymatic activity, suggesting that its antiviral action is due to the glycero-phospholipids cleavage on the virus envelope [67]. Glycerophospholipid hydrolysis would result in degradation of the virus envelope, leading to the exposure of the virus genomic RNA. The virus RNA degradation induced by RNase A after crotoxin and PLA_2_-CB treatment strongly supports this hypothesis. As degradation was considerably lower when compared to that induced by detergent (Triton X-100) and protease (proteinase K) treatments, these results suggest that PLA_2_-CB induces a partial envelope destabilization rather than full virus disassembly, in accordance with the PLA_2_-CB enzymatic function. This hypothesis is in agreement with the results obtained when *Newcastle disease virus* (NDV), an enveloped virus of the *Paramyxoviridae* family, was treated with PLA_2_s from different snakes [32]. NDV lost infectivity and cell fusing activity, which depends on envelope integrity, without significantly altering the hemagglutinin, hemolysin, or neuraminidase properties.

The fact that PLA_2_-CB inactivates DENV-2 via the cleavage of envelope glycerophospholipids, which are derived from host cell membranes [8], suggests that this enzyme could also exert some effect on other enveloped viruses, as we have already observed for *Yellow fever virus* [21]. This hypothesis was confirmed: PLA_2_-CB was able to inhibit other enveloped viruses such as *Rocio virus* from the same *Flaviviridae* family as DENV and YFV, *Oropouche virus* from the *Bunyaviridae* family, and *Mayaro virus* from the *Alphaviridae* family. Further support was revealed by the failure of the enzyme to inactivate the non-enveloped *Coxsackie B5 virus* from the *Picornaviridae* family.

The evidence that PLA_2_-CB inhibits virus infection via glycerol-phospholipid cleavage prompted us to determine how PLA_2_-CB gains access to the viral lipid-bilayer envelope if multiple copies of the E glycoprotein form a shell around it. A recent study about the extended configuration that DENV assumes at physiological temperatures revealed a series of gaps among the E proteins that provide an enhanced area of direct exposure of the virus lipid bilayer [40]. Although several channels can be identified on the E protein shell, allowing the communication between the virus lipid membrane and the environment, we focused on the pores found on each of the twenty 3-fold vertices of the virus surface. These pores, being the widest and deepest openings of the protein envelope, could provide the primary passageway for PLA_2_-CB monomers, which have been shown to be enzymatically active against glycerol-phospholipid substrates [68]. The size of the 3-fold axis pores with an area roughly twice as large as required by the enzyme's catalytic interface, provides a steric advantage to the accessibility of both PLA_2_-CB and crotoxin. The crotoxin complex (PLA_2_-CB and crotapotin) dissociates upon interaction with the membrane, and only the PLA_2_-CB unit binds to the substrate [69]. Accordingly, the area of contact for catalysis to occur is considered to be the same for crotoxin and PLA_2_-CB, which explains their similar efficiencies in virus inhibition.

Although sPLA_2_s show similar activity to phospholipids composed of different fatty acids, they do have particular affinities with regard to the phospholipid headgroup [70]. In addition, PLA_2_s containing tryptophan in the lipid-binding surface display the highest activity toward zwitterionic lipids [70]. PLA_2_-CB (Trp31, Trp70) can reportedly act upon both phosphatidylethanolamine (PE) and phosphatidylcholine (lecithin - PC), two major components of the DENV envelope, approximately 30 and 60% molar, respectively [44]. Several studies also confirm that PLA_2_-CB has a high affinity for lecithin-rich vesicles [71], [72], which justifies its synonym: lecithinase A or phosphatidylcholine 2-acylhydrolase [73], [74]. Furthermore, target membrane curvature has been attributed as an important modulating factor for PLA_2_ hydrolytic efficiency: the higher the curvature, the higher the enzymatic rate. Ahyayauch *et al.* [75] found increasing phosphatidylinositol-specific phospholipase C (PI-PLC) catalytic rates with decreasing curvature radii (increasing curvature) of unilamellar vesicles consisting of PI. Similarly, Berg et al. [76] showed that the fraction of total hydrolyzed substrate in the presence of pig pancreatic PLA_2_ changes from 50% to 71% as the size of unilamellar vesicles decreased. The results appear to be similar for venom phospholipases: Kensil and Dennis [77] described higher catalytic rates for cobra *Naja naja naja* PLA_2_ on small unilamellar PC vesicles (SUVs), with faster initial rates than those observed for large (LUVs) and multilamellar vesicles (MLVs). Interestingly, these authors detected distinctly higher catalytic rates on LUVs and MLVs at a small temperature range around the thermotropic phase transition of PC, at which a mixture of both gel and liquid crystalline phases are detected in vesicle lipid organization. Such a coexistence of two or more states triggers uneven phase boundary effects, which are loosely considered defects [78]. By observing phase boundary effects in monolayers, Grainger *et al.* [79] proposed that these prominences can be classified as defects; indeed, they provide a preferential site of action for PLA_2_s, as opposed to more homogeneous surface areas. Such preferential sites of action for PLA2s were also detected at monolayer borders, sites where the pronounced curvature provides effects similar to those of a disordered lipid patch [80]. In fact, Ahyayauch *et al.* [75] attributed the enhancement of catalytic rates to a successful partial penetration of the enzyme into the membrane hydrophobic matrix, which is more effortlessly achieved in regions of higher curvature and/or general defects on the bilayer surface. The DENV particle structure meets several of these PLA_2_ catalytic-enhancing conditions. Indeed, its diminutive size and spherical shape confer a relatively high curvature to its membrane (radius of curvature ∼232 Å) [40], characterizing only a marginally larger vesicle than a typical SUV, which, as already stated, provides a high-affinity substrate for PLA_2_ activity. Because of the described points of access to the bilayer on the DENV surface, PLA_2_-CB is able to hydrolyze the outer phospholipid layer, most likely accumulating fatty acids (FAs) and lysophospholipids (LPLs) on the membrane; this, in turn, destabilizes the membrane to a point at which fundamental protein conformational changes should be impaired. Additionally, it is known that the accumulation of LPLs in a bilayer causes an enhancement on its permeability [81], [82] and further activation of PLA_2_ [83], an observable fact with critical consequences to a supporting structure, such as a viral envelope. These modifications of the membrane structure allegedly disrupt the lipid order and thus its active liquid crystalline state, which most likely unbalances fundamental parameters such as the diffusion of anchored E proteins as well as their foothold, destabilizing the virus and its infective capability. Moreover, these disturbances are likely to drive the membrane to a highly disordered state, at which regions of bilayer breakdown can give rise to RNA exposure, which we detected with RNAse treatment and real-time RT-PCR.

In summary, we provide strong evidence that crotoxin and PLA_2_-CB have a direct effect on *Dengue virus* particles, most provably by glycerophospholipid cleavage on the virus envelope, which would lead to disruption of the lipid bilayer and destabilization of the E proteins on the virus surface, with the consequence of inactivation. Crotoxin and PLA_2_-CB most likely gain access to the *Dengue virus* lipid bilayer through the pores found on each of the twenty 3-fold vertices in the E protein shell on the virus surface. In addition, PLA_2_-CB was able to inactivate other enveloped viruses, highlighting its potential as a natural product lead for the development of broad-spectrum antiviral drugs.

## Supporting Information

Figure S1 **EC_50_ of crotoxin against DENV-2 in the virucidal assay.** DENV-2 (25–100 PFU) was incubated with serial decimal dilutions of crotoxin (50, 5, 0.5, 0.005, 0.0005, and 0.00005 ng/µL) for 1 h at 37°C. The treated viruses were used to infect VERO E6 cells contained in a 24-well plate. The percentage of inhibition was calculated by the reduction of the number of plaques found in the infected cells in comparison with the number of plaques in the cells infected with the untreated virus.
(TIF)

Figure S2 **EC_50_ of PLA_2_-CB against DENV-2 in the virucidal assay.** DENV-2 (25–100 PFU) was incubated with serial decimal dilutions of PLA2-CB (50, 5, 0.5, 0.005, 0.0005, and 0.00005 ng/µL) for 1 h at 37°C. The treated viruses were used to infect VERO E6 cells contained in a 24-well plate. The percentage of inhibition was calculated by the reduction of the number of plaques found in the infected cells in comparison with the number of plaques in the cells infected with the untreated virus.
(TIF)

Figure S3 **EC_50_ of crotoxin against ROCV in the virucidal assay.** ROCV (25–100 PFU) was incubated with serial decimal dilutions of crotoxin (50, 5, 0.5, 0.005, 0.0005, and 0.00005 ng/µL) for 1 h at 37°C. The treated viruses were used to infect VERO E6 cells contained in a 24-well plate. The percentage of inhibition was calculated by the reduction of the number of plaques found in the infected cells in comparison with the number of plaques in the cells infected with the untreated virus.
(TIF)

Figure S4 **EC_50_ of PLA_2_-CB against ROCV in the virucidal assay.** ROCV (25–100 PFU) was incubated with serial decimal dilutions of PLA_2_-CB (50, 5, 0.5, 0.005, 0.0005, and 0.00005 ng/µL) for 1 h at 37°C. The treated viruses were used to infect VERO E6 cells contained in a 24-well plate. The percentage of inhibition was calculated by the reduction of the number of plaques found in the infected cells in comparison with the number of plaques in the cells infected with the untreated virus.
(TIF)

Figure S5 **EC_50_ of crotoxin against MAYV in the virucidal assay.** MAYV (25–100 PFU) was incubated with serial decimal dilutions of crotoxin (50, 5, 0.5, 0.005, 0.0005, and 0.00005 ng/µL) for 1 h at 37°C. The treated viruses were used to infect VERO E6 cells contained in a 24-well plate. The percentage of inhibition was calculated by the reduction of the number of plaques found in the infected cells in comparison with the number of plaques in the cells infected with the untreated virus.
(TIF)

Figure S6 **EC_50_ of PLA_2_-CB against MAYV in the virucidal assay.** MAYV (25–100 PFU) was incubated with serial decimal dilutions of PLA_2_-CB (50, 5, 0.5, 0.005, 0.0005, and 0.00005 ng/µL) for 1 h at 37°C. The treated viruses were used to infect VERO E6 cells contained in a 24-well plate. The percentage of inhibition was calculated by the reduction of the number of plaques found in the infected cells in comparison with the number of plaques in the cells infected with the untreated virus.
(TIF)

Figure S7 **EC_50_ of crotoxin against OROV in the virucidal assay.** OROV (25–100 PFU) was incubated with serial decimal dilutions of crotoxin (50, 5, 0.5, 0.005, 0.0005, and 0.00005 ng/µL) for 1 h at 37°C. The treated viruses were used to infect VERO E6 cells contained in a 24-well plate. The percentage of inhibition was calculated by the reduction of the number of plaques found in the infected cells in comparison with the number of plaques in the cells infected with the untreated virus.
(TIF)

Figure S8 **EC_50_ of PLA_2_-CB against OROV in the virucidal assay.** OROV (25–100 PFU) was incubated with serial decimal dilutions of PLA_2_-CB (50, 5, 0.5, 0.005, 0.0005, and 0.00005 ng/µL) for 1 h at 37°C. The treated viruses were used to infect VERO E6 cells contained in a 24-well plate. The percentage of inhibition was calculated by the reduction of the number of plaques found in the infected cells in comparison with the number of plaques in the cells infected with the untreated virus.
(TIF)
